# Tumour‐associated antigenic peptides are present in the HLA class I ligandome of cancer cell line derived extracellular vesicles

**DOI:** 10.1111/imm.13471

**Published:** 2022-04-20

**Authors:** Pankaj Kumar, Caitlin Boyne, Sydney Brown, Ayesha Qureshi, Peter Thorpe, Silvia A. Synowsky, Sally Shirran, Simon J. Powis

**Affiliations:** ^1^ School of Medicine University of St Andrews St Andrews UK; ^2^ School of Biology University of St Andrews St Andrews UK; ^3^ Biomedical Sciences Research Complex University of St Andrews St Andrews UK

**Keywords:** breast cancer, extracellular vesicles, HLA ligandome, T‐cell epitopes, tumour associated antigen (TAA)

## Abstract

The recent success of monoclonal antibody checkpoint inhibitor therapies that enhance the ability of CD8^+^ T cells to detect cancer‐related antigenic peptides has refocused the need to fully understand the repertoire of peptides being presented to the immune system. Whilst the peptide ligandome presented by cell surface human leucocyte antigen class I (HLA‐I) molecules on cancer cells has been studied extensively, the ligandome of extracellular vesicles (EVs) remains poorly defined. Here, we report the HLA‐I ligandome of both the cell surface and EVs from eight breast cancer cell lines (MCF7, MDA‐MB‐231, MDA‐MB‐361, MDA‐MB‐415, MDA‐MB‐453, HCC 1806, HCC 1395, and HCC 1954), and additionally the melanoma cell line ESTDAB‐056 and the multiple myeloma line RPMI 8226. Utilizing HLA‐I immunoisolation and mass spectrometry, we detected a total of 6574 peptides from the cell surface and 2461 peptides from the EVs of the cell lines studied. Within the EV HLA‐I ligandome, we identified 150 peptides derived from tumour associated antigenic proteins, of which 19 peptides have been shown to elicit T‐cell responses in previous studies. Our data thus show the prevalence of clinically relevant tumour‐associated antigenic peptides in the HLA‐I ligandome presented on EV.

## Graphical abstract

The HLA‐I peptidomes of both EV and cell were identified from 10 cancer cell lines and 150 peptides derived from tumour‐associated antigens were identified in HLA‐I ligandome of EVs. Our data, show, for the first time the prevalence of clinically relevant tumour‐associated antigenic peptides in the HLA‐I ligandome presented on EV and the potential of EV ligandomes for identification of CD8^+^ T‐cell epitopes.

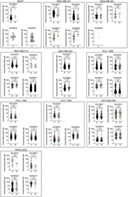

**Abbreviations**
CL: cell lysates
EVs: extracellular vesicles
HLA‐I: human leucocyte antigen class I
TAA: tumour‐associated antigenic proteins
TAApep: peptides derived from tumour‐associated antigenic proteins

## INTRODUCTION

Human leucocyte antigen class I molecules (HLA‐I) display short peptides, typically 9–11 amino acids in length, primarily derived from the cellular proteome, to CD8^+^ T cells which permits the detection of intracellular pathogens such as viruses, and also up‐regulated or mutated proteins relevant in cancer [1] However, a key problem in the context of cancer cells is their ability to evade elimination by cytotoxic CD8^+^ T cells through the expression of immune checkpoint proteins such as PD‐L1. In recent years, checkpoint inhibitor therapies in the form of monoclonal antibodies directed against PD‐L1 and PD1 have displayed significant clinical success by allowing CD8^+^ T cells to more readily detect the array of antigenic peptides on cancer cells [2, 3, 4]. This in turn has led to enhanced efforts to fully describe the cancer HLA‐I peptide proteome in ever more detail [5]. Such efforts have naturally concentrated on the cell surface HLA‐I ligandome. However, the nature of the HLA‐I ligandome present on extracellular vesicles (EVs) released by cancer cells remains essentially unknown.

Extracellular vesicles, including exosomes, are typically 50–200 nm membrane bound vesicles secreted by cells into extracellular environment [6]. EVs released into the extracellular space can carry bioactive molecules including RNAs (mRNA, microRNA, and long non‐coding RNA), lipids, and proteins. Depending on their origin and biological information carried, EVs can instigate changes in the recipient cells according to the information transferred [7]. Once EVs have entered the extracellular space or circulation, they are thought to elicit effects by one of two mechanisms: by fusing with the plasma membrane of recipient cells, resulting in the release of their contents to the cell [8], or second, by direct interaction with receptors on the surface of the target cells, initiating the activation of various signalling pathways [9]. EVs have been proposed as key players in cell communication, and in particular the communication that exists between cancer cells and the host microenvironment, both locally and at a distant site [10]. EVs derived from many cancer cells or within the tumour microenvironment are thought to be key factors in oncogenic transformation, drug resistance, and tumour metastasis. On the other hand, EVs are also involved in tumour rejection [11] indicating that the role of EVs in tumour progression relies on their cargo. Therefore, defining the EV cargo is crucial to fully understand the role of EVs in tumour growth or rejection. Additionally, tumour‐derived EVs or exosomes (TEX) have been proposed as cancer prognosis markers for a range of tumours [12, 13]. For example, TEX and T‐cell derived EVs were used for monitoring head and neck cancer patients' response to oncology therapy [14].

It has been known for many years that tumour‐derived EVs are present in malignant effusions and that these EVs are enriched in HLA molecules as well as tumour antigens such as *HER2/neu* and melan‐A [15]. Additionally, EVs derived from tumour cells can modulate antigen specific CD8+ T‐cell response either by direct presentation or cross‐presentation, indicating that EVs carry functional HLA‐peptide complexes [16, 17]. However, the actual HLA‐I ligandome of EVs released by tumour cells and its potential in identifying tumour antigens (TAs), has not been studied extensively. We hypothesized that defining the HLA‐I ligandome of EVs released by tumour cells may potentially be utilized to identify clinically relevant tumour antigens. This hypothesis is based on our previous finding that the HLA‐I ligandome of EVs is similar to the cell ligandome [18]. Here, we report the results of a study aimed at the identification of EV HLA‐I ligandomes, and more crucially their potential in identification of tumour antigens, from breast cancer, melanoma, and myeloma cell lines.

## EXPERIMENTAL PROCEDURES

### Cell lines and flow cytometry

The breast cancer cell lines, MDA‐MB‐231, MDA‐MB‐361, MDA‐MB‐415, MDA‐MB‐453, MCF7, HCC 1395, HCC 1806, and HCC 1954, were obtained from the ATCC Breast Cancer Cell panel (ATCC 30‐4500K, LGC Standards). Multiple myeloma cell line RPMI 8226 was obtained from LGC Standards and melanoma cell line ESTDAB‐056 was a gift from Prof Federico Garrido, (University of Granada, Spain). All cell lines were cultured in DMEM or RPMI‐1640 supplemented with 5% fetal bovine serum (FBS) and 50 μg/ml kanamycin (all ThermoFisher Scientific). For flow cytometry, cells were harvested from culture flasks with a 1–2 min incubation with trypsin (0.025%)‐EDTA (0.01%) solution at 37° celsius. Cells were then resuspended in culture medium to inactivate trypsin and centrifuged at 300*g* for 10 min at 4° celsius, then resuspended in PFN buffer (PBS, 2% FBS, 0.1% sodium azide). Cells were stained with primary mouse IgG anti‐HLA‐A, ‐B, and ‐C monoclonal antibody W6/32 [19] for 20 min at 4° celsius, followed by two washes with centrifugation steps as above in PFN. Cells were then stained with FITC‐anti‐mouse IgG (Sigma‐Aldrich UK, F2012) at a dilution of 1/100 for 20 min, and washed as above. Control cells received second stage FITC anti‐mouse IgG alone. Cells resuspended in PFN and were analysed on a Merck‐Millipore Guava 8HT flow cytometer with a 488‐nm laser using Guavasoft 2.7 software.

### Characterization of EV

For the initial characterization of EVs, cell lines were grown in serum‐free medium (Ex‐Cell 610 HSF serum free, Sigma‐Aldrich, UK) to exclude the possibility of EV contamination from serum. For nanoparticle tracking analysis (NTA), cells were cultured for 24 hr, conditioned media were then collected and centrifuged at 300*g* for 10 min at 4° celsius to remove cells and large debris. EV‐containing supernatants were then filtered with 0.22‐μm Millex‐GP syringe filters (Merck). NTA was performed using an LM‐10 unit (Malvern) equipped with a 638‐nm laser. A minimum of three videos of 30 s were recorded for each sample using settings of shutter speeds of 17 or 30 ms. Data analysis was performed using NTA 2.3 software with detection thresholds of 2 or 3 and blur, min track length, and min expected size on auto settings. One representative data plot is shown for each cell line.

For immunoblotting, cells were grown in 175 cm^2^ flasks for 48 hr in serum‐free medium as above. After 48 hr, conditioned media were collected, spun, and filtered through 0.22‐μm filters as above, and culture supernatants were then spun at 100 000*g* for 2 hr using a Sw32Ti swing‐out rotor in a Beckman L100 ultracentrifuge. EV pellets were resuspended in lysis buffer (1% NP40, 150 mM NaCl, 10 mM Tris pH 7.4, supplemented with Pierce mini‐protease inhibitor tablets). Cell lysates were prepared concomitantly by lysis of the cells remaining in the culture flasks with the same lysis buffer, with an additional spin at 20 000*g* for 10 min at 4° celsius to remove insoluble debris. Protein estimations were performed by Bradford assay (ThermoFisher Scientific). Approximately, 3 μg of cell and EV lysates were electrophoresed on 4%–20% gradient sodium dodecyl sulphate‐polyacrylamide gel electrophoresis gels (ThermoFisher Scientific) and transferred to nitrocellulose filters (BA85, ThermoFisher Scientific). Membranes were probed with anti‐CD9, CD63, and CD81 antibodies (ThermoFisher Scientific clones Ts9, Ts63, and M38, respectively) at 1:5000 dilution, anti‐HLA‐B and ‐C mouse monoclonal antibody HC10 [20] at 1:1000 dilution, anti‐human TAP1 (Merck, clone 148.1) at 1:1000 dilution, or rabbit monoclonal anti‐human calnexin (Abcam, ab213243) at 1:1000 dilution. Immunoblot signals were revealed with 1:10 000 diluted IR Dye800cw anti‐mouse or anti‐rabbit IgG (LI‐COR) and visualized using a LI‐COR Odyssey scanner.

### Large‐scale EV and cell isolation for HLA‐I peptide isolation

Large‐scale cultures for EV and cell isolation were maintained in EV‐depleted FBS. EV‐depleted FBS was prepared by ultracentrifugation of FBS at 100 000*g* for 4 hr at 4° celsius using a SW32Ti rotor in a Beckman L100 ultracentrifuge, followed by 0.2‐μm filtration. All cell lines were either grown in DMEM (breast cancer cell lines) or in RPMI‐1640 (myeloma and melanoma cell lines) supplemented with 2.5% EV‐depleted FBS and 50 μg/ml kanamycin. Suspension cell line RPMI 8226 was cultured in 175‐cm^2^ flasks and cultured to approximately 2 million cells per ml. Conditioned medium containing cells and EVs was collected and centrifuged at 300*g* for 10 min at 4° celsius. The supernatant containing EVs was filtered with 0.2‐μm syringe filter and stored at −20° celsius and RPMI 8226 cells were re‐plated in EV‐depleted medium. Conditioned medium was collected again after 48 hr and processed as described above.

All adherent cells were grown in either multiple 175‐cm^2^ flasks or in Falcon 5‐layer multi‐flasks with a surface area of 875 cm^2^ (ThermoFisher Scientific). At around 80% cell confluence, fresh culture medium was added to flasks and conditioned medium was harvested every 48 hr and processed as above, with new medium then added to the flasks. In total 800 ml conditioned media were harvested from each breast cancer cell line and 250 ml conditioned medium was harvested from ESTDAB‐056 and suspension cell line RPMI 8226. The EV harvests were thawed and spun at 100 000*g* for 2 hr using a Sw32Ti rotor and the EV pellet lysed in lysis buffer as above, and stored at −20° celsius until immunoprecipitation. At culture closedown, cells were harvested from culture flasks with a 1–2 min incubation with trypsin (0.025%)‐EDTA (0.01%) solution at 37° celsius and spun at 300*g* for 10 min at 4° celsius. Cell pellets were lysed in lysis buffer as above, spun at 20 000*g* for 10 min at 4° celsius to remove debris and the cell lysates were stored at −20° celsius until immunoprecipitation. EV and cell isolation experiments were independently repeated two to three times for breast cancer cell lines and six times for myeloma and melanoma cell lines.

### Immunoprecipitation and mass spectrometry analysis

EV and cell lysates (CL) were thawed and incubated with 0.5 ml anti‐HLA‐A, ‐B, and ‐C antibody, W6/32, coupled to Protein G‐Sepharose beads (crosslinked to Protein G using BS3 crosslinker according to manufacturer instructions, ThermoFisher Scientific) for 1–2 hr. The beads were then washed extensively with wash buffer (150 mM NaCl, 10 mM Tris pH 7.4), and resuspended in 1 ml of 1% TFA for 10 min at room temperature to release HLA‐I and bound peptides. Eluted HLA‐I and peptides were then bound to Pierce C18 100‐μl tips (87784, ThermoFisher Scientific) based on the manufacturer's instructions. The peptide fraction was eluted in 30% acetonitrile and 0.1% TFA and dried down by speedvac for mass spectrometry.

Peptides were then analysed on an AB Sciex TripleTOF 5600+ system mass spectrometer (Sciex) coupled to an Eksigent nanoLC AS‐2/2Dplus system. The samples were loaded in loading buffer (2% acetonitrile and 0.05% trifluoroacetic acid) and bound to an Aclaim pepmap 100 μm × 2 cm trap (ThermoFisher Scientific), and washed for 10 min to waste after which the trap was turned in‐line with the analytical column (Aclaim pepmap RSLC 75 μm × 15 cm). The analytical solvent system consisted of buffer A (2% acetonitrile and 0.1% formic acid in water) and buffer B (2% water with 0.1% formic acid in acetonitrile) at a flow rate of 300 nl/min with the following gradient: linear 1%–20% of buffer B over 90 min, linear 20%–40% of buffer B for 30 min, linear 40%–99% of buffer B for 10 min, isocratic 99% of buffer B for 5 min, linear 99–1% of buffer B for 2.5 min, and isocratic 1% solvent buffer B for 12.5 min. The mass spectrometer was operated in the DDA top 20 positive ion mode, triggering on +2 to +5 charge states, with 120 and 80 ms acquisition time for the MS1 (*m*/*z* 400–1250) and MS2 (*m*/*z* 95–1800) scans, respectively, and 15‐s dynamic exclusion. Rolling collision energy was used for fragmentation.

### Peptide identification

LC–MS/MS data were searched against the human proteome (uniprot database UP000005640 containing 74 464 protein entries, downloaded on 2 August 2019) with an additional contaminant list from the global proteome machine (cRAP protein sequences). We used decoy fusion method of PEAKS DB search to estimate false discovery rate during peptide identification [21]. Peptide identification was performed with PEAKS Studio X (https://www.bioinfor.com/peaks-studio). Following mass spectrometry data acquisition, data files from the AB Sciex Triple TOF 5600+ were converted into mzML format using MSConvert software of proteowizard. Converted data files were imported into PEAKS Studio X for peptide identification. Data files were subjected to default data refinement followed by PEAKS de novo and PEAKS DB (database) searches to identify peptide sequences. PEAKS de novo and PEAKS DB searches were carried out by setting parent mass error tolerance to 15 parts per million (ppm), the fragment mass error tolerance to 0.1 Da, no enzyme selection, unspecific digestion mode, and filtering charge between 2 and 5. No post‐translational modification (PTM) was selected in PEAKS de novo and PEAKS DB searches. A 0.1% FDR cut off was applied during PEAK DB searches to select high confidence peptides and peptides only identified by PEAKS DB were selected for further analysis.

Mass spectrometry proteomic data have been deposited to the ProteomeXchange Consortium via the PRoteomics IDEntifications (PRIDE) repository with the dataset identifier PXD025345.

### HLA typing of cell lines

The HLA‐I genotypes of breast cancer cell lines, MDA‐MB‐231, MDA‐MB‐361, MDA‐MB‐415, MDA‐MB‐453, HCC 1806, HCC 1395, and HCC 1954, were identified by using paired end reads in fastq format as input to the Optitype version 1.3.1 [22]. Scripts used to perform HLA‐I typing can be found on: https://github.com/peterthorpe5/Cancer_cell_line_RNAseq_assemblies. RNA‐seq data of breast cancer cell lines were obtained from the Gene Expression Omnibus (GEO) (accession GSE73526). The HLA‐I genotypes of breast cancer cell line, MCF7, was obtained from previously reported HLA‐I genotypes of cancer lines [23]. HLA‐I genotype of ESTDAB‐056 was identified from immune polymorphism database (https://www.ebi.ac.uk/cgi-bin/ipd/estdab/print_cell.cgi?ESTDAB-056) and RPMI 8226 HLA‐type was determined from previous report [24]. HLA‐I types of all cancer cell lines are presented in File S1.

### HLA‐I peptide binding affinity and data analysis

Eluted peptides identified from each replicate were combined, duplicate peptides were removed and filtered to select 8–15 amino acid long peptides. The total number of 8‐15‐mer peptides was defined as HLA‐I ligandome of cell and EVs from each cell line. Peptides present in HLA‐I ligandome were assessed for their predicted allele‐binding and affinity using algorithm netMHCpan 4.0 (http://www.cbs.dtu.dk/services/NetMHCpan/) [25] following default settings. Peptides with IC_50_ up to 1000 nM were tabulated and plotted using Prism 8 (GraphPad, Inc.) software. For identification of immunogenic peptides reported in previous studies and the peptides derived from TAA proteins, peptides with 8–15 length were searched against IEDB database (https://www.iedb.org/database_export_v3.php; tcell_full_v3_zip) and Tantigen database (http://projects.met-hilab.org/tadb/index.php).

## RESULTS

### Characterization of cell lines and extracellular vesicles

**FIGURE 1:**
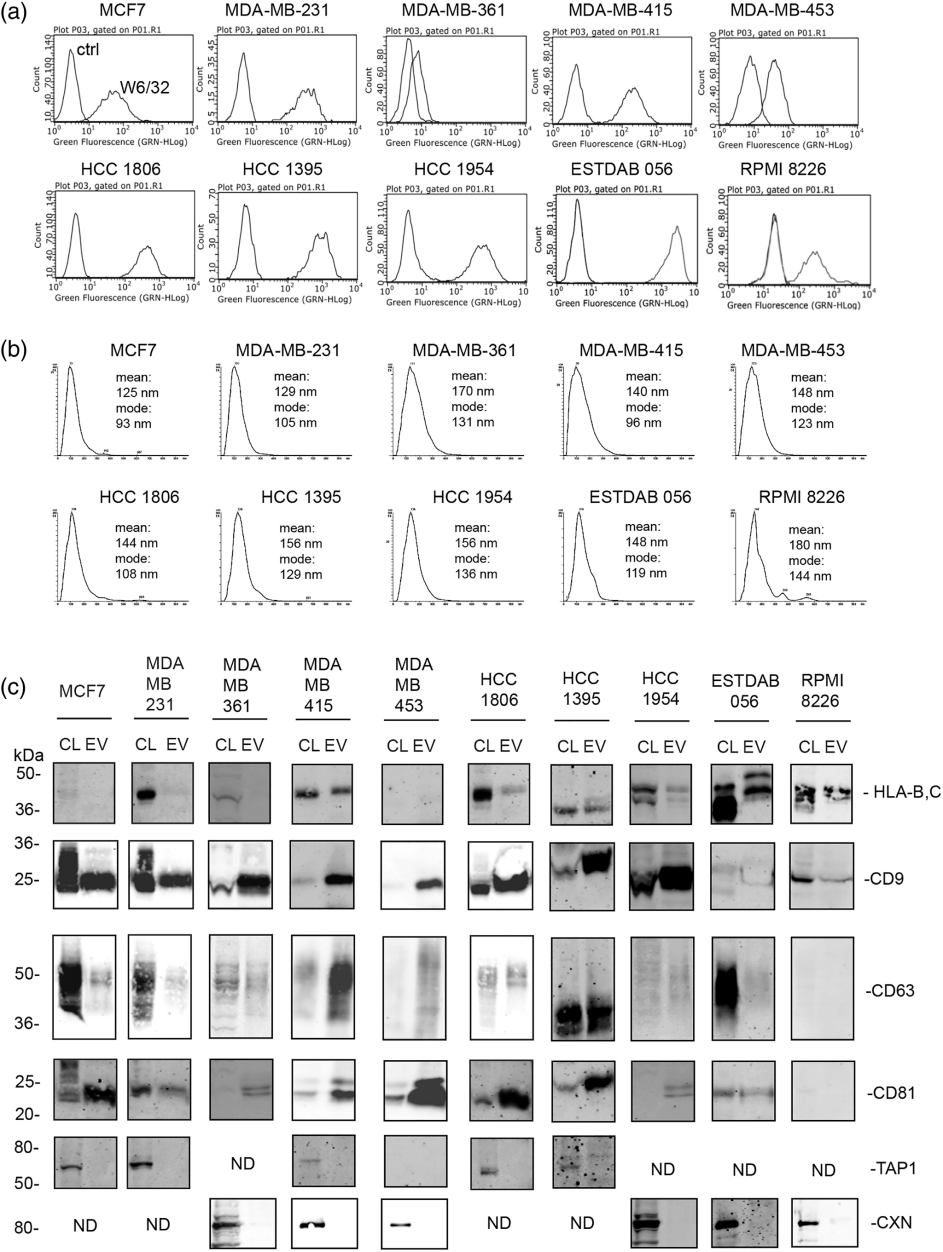
(a) Flow cytometry of cell surface HLA‐A, B, and C as detected by antibody W6/32 compared to control second‐stage FITC anti‐IgG alone (ctrl). (b) NTA analysis of particles released by cell lines. One comparative graph of three recordings is shown, with mean and modes indicated. (c) Immunoblotting analysis of detergent cell lysates (CL) and EV lysates of all cell lines, probed for HLA‐B and C, CD9, CD63, CD81, and control proteins transporter associated with antigen processing (TAP1) and calnexin (CXN)

We first characterized the expression of HLA‐I molecules on our chosen cell lines. Cell surface flow cytometry was performed on the breast cancer cells lines MCF7, MDA‐MB‐231, MDA‐MB‐361, MDA‐MB‐415, MDA‐MB‐453, HCC 1806, HCC 1395, and HCC 1954, the melanoma cell line ESTDAB‐056, and the myeloma cell line RPMI 8226, using the HLA‐A, B, and C specific monoclonal antibody W6/32 (Figure 1a). HLA‐I expression levels varied, with cell line MBA‐MB‐361 displaying the lowest apparent levels and ESTDAB‐056 displaying the highest levels of relative expression, but overall, all of the cell lines expressed cell surface HLA‐I molecules.

To characterize the EVs released by each of these cell lines and to determine HLA‐I expression on EVs, cell lines were cultured in serum‐free media to eliminate potential contamination from FBS‐derived EVs. We performed nanoparticle tracking analysis (NTA) on conditioned medium collected from cells grown for 24 hr in these serum‐free conditions (Figure 1b). All cell lines released particles that were detected by NTA with means and modes that were within the 90–200 nm range. We also performed immunoblot analysis on cell lysates (CL) and EV lysates (EV isolated by filtration and ultracentrifugation) from cultures grown in serum‐free conditions for 48 hr (Figure 1c). The expression of characteristic markers for exosomes and EVs varied, but all EV lysates from breast cancer lines were positive for CD9 and CD81, whilst the presence of CD63 was more variable. The cell and EV lysates from myeloma line RPMI 8226 was positive for CD9 but negative for CD63 and CD81. The EV lysate from melanoma line ESTDAB‐056 showed a high signal for CD9 (image saturated in Figure 1c) but was low for CD63 and CD81. EV lysates from all lines were negative for the control endoplasmic reticulum proteins TAP1 or calnexin (CXN) indicating EV lysates were devoid of contaminating cellular components. The detection of HLA‐I molecules was variable, with cell lines such as MDA‐MB‐415, ESTDAB‐056, and RPMI 8226 displaying strong signals. Lower HLA‐I signals were observed in cell lines, HCC 1806, HCC 1395, and HCC 1954. HLA‐I signal was not detected in cell lines MCF‐7, MDA‐MB‐361, MDA‐MB‐231, and MDA‐MB‐453. It should be noted however that the monoclonal antibody HC10 used in these immunoblots reacts only with the heavy chain of HLA‐B and C molecules. Therefore, HLA‐A molecules will be under‐reported in these data. Taken together, we characterized our vesicle preparation as typical of a population described as extracellular vesicles based on MISEV guidelines [26].

### HLA‐I ligandome of cells and EVs

Based on our previous study [18], we used a protocol that promotes speed of isolation and processing of both CL and EV lysates, to reduce the risk that lower affinity peptides bound by HLA‐I molecules would be lost due to HLA‐I protein unfolding. Thus, cell culture supernatants grown in EV‐depleted serum containing medium were centrifuged at 300 x *g* to remove debris, filtered at 0.2 μm and then ultracentrifuged at 100 000*g* for 2 hr to isolate EV, followed by detergent lysis and immunoisolation with antibody W6/32 coupled to Sepharose beads. CLs were processed concomitantly. After release of the peptides by acidification in trifluoracetic acid and clean up on C18 matrix tips, peptides were analysed by mass spectrometry. HLA‐I immunoisolated and eluted peptides were identified by PEAKS DB software (hereinafter defined as eluted peptides). Eluted peptides from independent experiments of cell and EV lysates of each cell line were combined together and duplicate peptides (peptides identified in more than one experiment) were removed to obtain a complete list of unique eluted peptides from cell or EVs. Peptides which are 8–15 amino acids long have been reported to bind HLA‐I molecules and elicit immune responses. Therefore, eluted peptides identified by PEAKS DB, were filtered to select 8‐15‐mer peptides for further analysis. The total number of 8‐15‐mer peptides identified from cell and EV lysates is hereinafter defined as cell or EV ligandome, respectively.

The details of eluted peptides and HLA‐I ligandomes from each cell lines are presented in File S2 and summarized in Tables 1 and 2. A total of 6574 and 2461 eluted peptides were identified from cell and EVs of all 10 cell lines, respectively (Column 2 of Tables 1 and 2). A total of 6144 and 2406 peptides were identified as the cell and EV ligandomes from all 10 cell lines, respectively (Column 3 of Tables 1 and 2). The highest number of peptides were identified in ligandomes of cell lines, HCC 1954, HCC 1806, ESTDAB‐056, MDA‐MB‐415, and HCC 1395 (Column 3 of Tables 1, 2 and File S2), which is in agreement with the flow cytometry data of HLA‐I expression on cell surface (Table 1 and Figure 1a). A similar correlation was observed between HLA‐I expression detected in immunoblots and number of peptides in EV ligandomes of different cell lines. For example, expression of HLA‐I was not detected/below detection with mAb HC10 in immunoblots of EVs of MCF7, MDA‐MB‐231, MDA‐MB‐361, and MDA‐MB‐453 (Figure 1c) which are the cell lines with least number of peptides in their respective EV ligandomes (Column 3 of Table 2 and File S2). The cell and EV ligandomes were also compared with each other to determine the proportion of peptide common between cell and EVs. To facilitate this analysis, peptides from cell ligandomes of all cell lines were combined and duplicate peptides i.e., the same peptide identified from more than one cell line, were removed. Similarly, peptides of EV ligandomes were combined and duplicate peptides were removed before comparing the HLA‐I ligandomes of cell and EVs (File S3). A total of 5503 and 2244 unique peptides were found to constitute the cell and EV ligandomes of all 10 cell lines, respectively. Of 2244 peptides found in the EV ligandome, 74% peptides were also detected in the cell ligandome. However, 26% (589) peptides of the EV ligandome were not detected in cells (Figure S1 and File S3). A similar observation, albeit varying proportion of common and unique peptides, was made when peptides of cell and EV ligandomes were compared separately for breast cancer, myeloma and melanoma cell lines (Figure S1 and File S3).

**TABLE 1:** Details of total eluted peptides, HLA‐I ligandome, netMHCpan4.0 predicted binding peptides, previously reported T‐cell epitopes and peptides derived from TAA proteins from the cell surface of breast cancer, melanoma and myeloma cell lines

1. Cell lines	2. Number of eluted peptides	3. Number of peptides in HLA‐I ligandome (8‐15‐mer peptides)	4. Number of netMHCpan 4.0 binding peptides	5. Number of known immunogenic peptides identified in the study	6. Number of peptides derived from known TAA (TAApep)
MCF7	625	461	214	11	22
MDA‐MB‐231	412	406	359	11	25
MDA‐MB‐361	178	168	134	0	9
MDA‐MB‐415	827	818	700	2	52
MDA‐MB‐453	621	569	453	3	18
HCC 1806	873	756	633	6	36
HCC 1395	727	693	606	2	39
HCC 1954	842	828	720	3	44
ESTDAB‐056	804	795	717	4	40
RPMI 8226	665	650	580	1	37
Total	6574	6144	5116	43	322

**TABLE 2:** Details of total eluted peptides, HLA‐I lignadome, netMHCpan4.0 predicted binding peptides, previously reported T‐cell epitopes and peptides derived from TAA proteins from the EV of breast cancer, melanoma, and myeloma cell lines

1. Cell lines	2. Number of eluted peptides	3. Number of peptides in HLA‐I ligandome (8‐15‐mer peptides)	4. Number of netMHCpan 4.0 binding peptides	5. Number of known immunogenic peptides identified in the study	6. Number of peptides derived from known TAA (TAApep)
MCF7	43	18	5	2	0
MDA‐MB‐231	38	38	32	1	0
MDA‐MB‐361	67	67	35	1	4
MDA‐MB‐415	336	336	275	2	19
MDA‐MB‐453	127	120	115	2	5
HCC 1806	363	345	306	3	18
HCC 1395	534	533	502	2	32
HCC 1954	331	330	293	2	23
ESTDAB‐056	472	472	433	3	24
RPMI 8226	150	147	112	1	6
Total	2461	2406	2108	19	131

**FIGURE 2:**
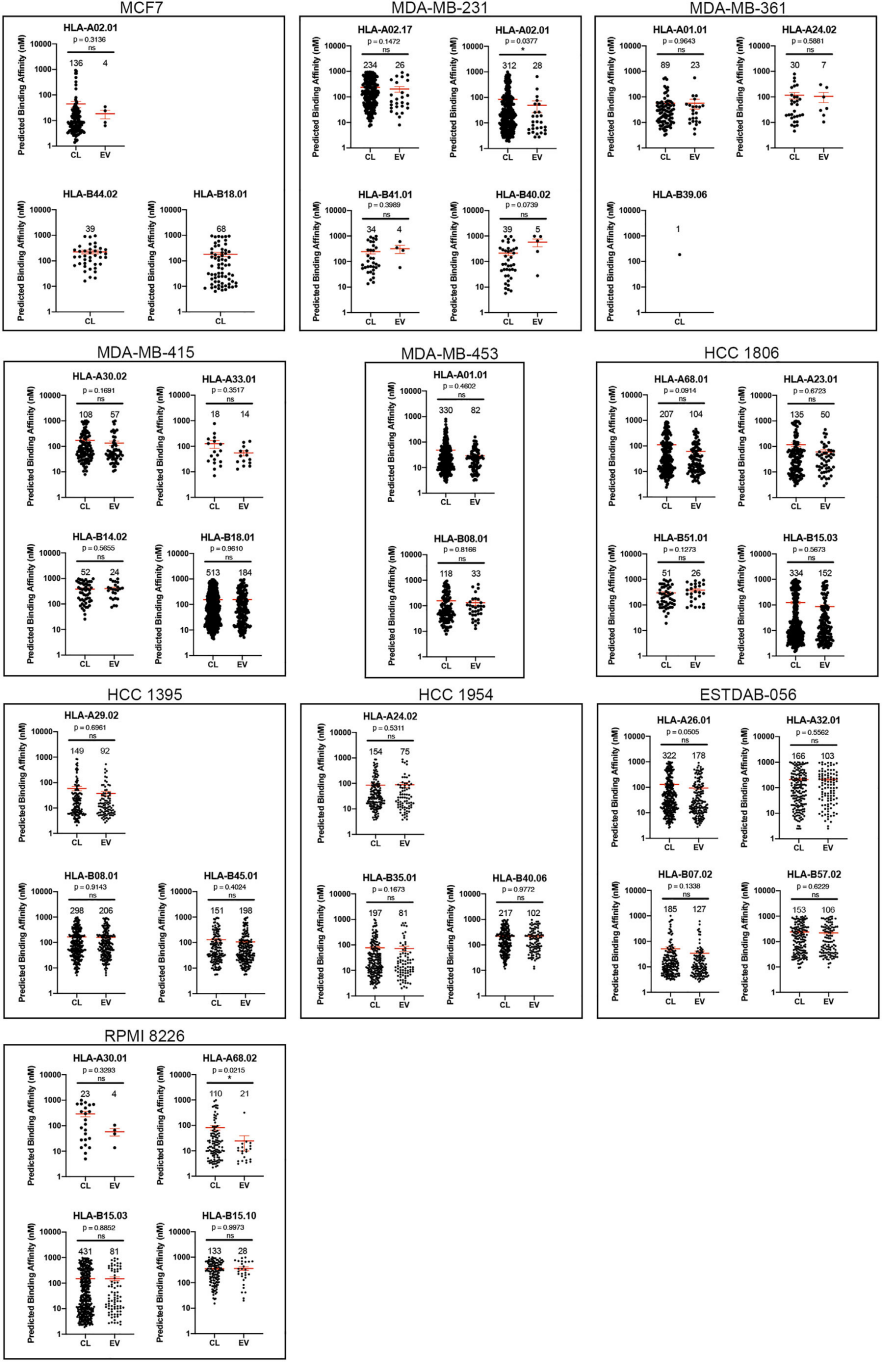
Predicted HLA‐A‐ and B‐binding affinities of peptides from cells (CL) and EV, determined using algorithm netMHCpan 4.0. Each dot represents a single identified peptide. The numbers above each plot indicates the number of ligands for each respective HLA‐I allele. Two‐tailed Mann–Whitney test was performed, with mean predicted affinity ± SE shown in red. ns, not significant; *p < 0.05

Peptides present in HLA‐I ligandomes were further processed through the HLA‐I binding affinity prediction algorithm netMHCpan 4.0. Details of HLA‐I binding peptides from each cell line, for each expressed HAL‐I allele, and their respective predicted binding affinities (BA) are presented in Figures 2 (HLA‐A and HLA‐B) and S2 (HLA‐C). Predicted mean binding affinities are presented in Table S1. It was noted that the mean predicted binding affinity of peptides for HLA‐A molecules was frequently lower (strong binding) in the EV pool compared to the cell surface pool (shown in Table S1). Two‐tailed Mann–Whitney tests indicated no significant differences in HLA‐I binding affinities between the cell and EV peptidomes, except in cases such as HLA‐A*2:01 in MDA‐MB‐231, HLA‐A*68:02 in RPMI 8226 (Figure 2). However, low numbers of peptides in the EV peptidome limits the interpretation of significance values in these samples. In addition, it was noted that the HLA‐I binding affinity predictions for alleles A*02:01 and A*02:17 in MDA‐MB‐231 were very distinct from each other. These alleles differ at key positions 95, 97, and 99 in the middle of the HLA‐I peptide‐binding groove which will likely impact on peptide selections. Peptide ligands from EVs and cells were also compared to determine peptide length distribution and sequence motifs. As reported previously [18], we also found that peptide from cells and EVs distributed similarly in length and were composed of peptides with similar sequence motifs (data not shown).

### Identification of peptides derived from tumour‐associated antigens and putative immunogenic peptides

Owing to the presence of HLA‐I molecules, EVs have long been suspected to carry tumour antigens. A detailed description of the peptidome and the full extent and identity of tumour antigens has, however, not been reported for EVs. Therefore, EV ligandomes of each cell line were searched for the presence of tumour antigens. For clarity, herein we define TAA as tumour‐associated antigenic proteins and TAApep as the peptides derived from tumour‐associated antigenic proteins that are presented on HLA‐I molecules.

First, we determined if EVs can carry immunogenic peptides which have been identified in previous studies. The cell and EV ligandomes were searched for the presence of peptides showing sequence match to known T‐cell epitopes using IEDB and Tantigen databases since IEDB and Tantigen databases are currently amongst the most comprehensive tumour antigen databases. The number of identified peptides showing sequence match with known T‐cell epitopes from EV and cell ligandomes are presented in Tables 1 and 2. A total of 43 peptides from cell ligandomes were identified to match 31 known T‐cell epitopes (Table S3; Table 1, Column 5). In EV ligandomes, 19 peptides were found to match with 15 known T‐cell epitopes (Table 2, Column 5; Table 3). Importantly, known T‐cell epitope sequences were detected in the EV ligandomes of all cell lines (Tables 2 and 3) including MCF7, MDA‐MB‐361, and MDA‐MB‐231 where only a limited number of peptides were identified in the EV ligandome (Table 2). For example, in the EV ligandome of MCF7, only 18 peptides were identified, but amongst them were two known T‐cell epitope sequences (NTDSPLRY and ALSDHHIYL, Table 3). The peptide NTDSPLRY is derived from the 40S ribosomal protein SA, an oncofetal antigen expressed by tumours [27].

**TABLE 3:** List of peptides matching with previously identified T‐cell epitopes from EV HLA‐I ligandomes of breast cancer, melanoma, and myeloma cell lines

Number	Antigen name	Parent protein	Epitope ID	Position	Uniprot entry name	PubMed ID	T‐cell epitope	HLA ‐I restriction	Peptides identified in the study	Cell line
1	Carcinoma‐associated mucin	P15941	T000434	130–138, multiple positions	MUC1_HUMAN	10361129	STAPPAHGV	HLA‐A*02:01, HLA‐A*11:01	STAPPAHGV	RPMI 8226
2	Ubiquitin‐conjugating enzyme E2 D2	P62837	243 855	59–66	UB2D2_HUMAN	22869377	YPFKPPKV	HLA‐B*51	YPFKPPKVTF*	HCC 1954
									YPFKPPKV	HCC 1806
3	Chromatin Assembly Factor 1 Subunit A	Q13111	T001077	772–781	CAF1A_HUMAN	24048523	SPRSPSTTYL	HLA‐B*07:02	SPRSPSTTYL	ESTDAB‐056
4	Clathrin heavy chain 1	Q00610	442 923	311–320	CLH1_HUMAN	22869377	ATAGIIGVNR	HLA‐A*11	ATAGIIGVNR	HCC 1806
5	G1/S‐specific cyclin‐D1	P24385	T000697	115–124	CCND1_HUMAN	12384544	ETIPLTAEKL	HLA‐A*68:01	ETIPLTAEKL	HCC 1806
6	Cytochrome P450 1B1	Q16678	T000701	5–13	CP1B1_HUMAN	12869499	FLDPRPLTV	HLA‐A*02:01	FLDPRPLTV	MDA‐MB‐231
7	E3 ubiquitin‐protein ligase Mdm4	Q00987	236 802	273–281	MDM2_HUMAN	25548167	DEVYQVTVY	HLA‐B*18	DEVYQVTVY	MDA‐MB‐415
8	Fructose‐bisphosphate aldolase A	P04075	2874	216–224	ALDOA_HUMAN	11782012	ALSDHHIYL	HLA‐A*02:01	ALSDHHIYL	MCF7
9	Histone H3.3	P84243	T000946	59–67	H33_HUMAN	16196104	ELLIRKLPF	HLA‐B*08	ELLIRKLPF	HCC 1395
									ELLIRKLPF	MDA‐MB‐453
10	HER2 receptor	P04626	67 385	63–71	ERBB2_HUMAN	17397516	TYLPTNASL	HLA‐A*24	TYLPTNASLSF*	HCC 1954
11	Mammaglobin‐A	Q13296	64 399	32–40	SG2A2_HUMAN	15538043	TINPQVSKT	HLA‐A*02	KTINPQVSKTEY*	MDA‐MB‐415
12	Poly (ADP‐ribose) polymerase family, member 12	Q9H0J9	T000954	669–677	PAR12_HUMAN	16033845	VYPEYVIQY	HLA‐C*07:02	VYPEYVIQY	ESTDAB‐056
13	Poly(RC) Binding Protein 2	Q15366	T001070	183–191	PCBP2_HUMAN	24048523	RPKPSSSPV	HLA‐B*07:02	RPKPSSSPVIF*	ESTDAB‐056
14	Similar to retinoblastoma‐binding protein 4, partial	Q09028	215 983	245–253	RBBP4_HUMAN	22869377	NLKLKLHSF	HLA‐B*57	NLKLKLHSF	HCC 1395
15	40S ribosomal protein SA	P08865	T000873	146–154	RSSA_HUMAN	16709854	ALCNTDSPL	HLA‐A*02:01	NTDSPLRY*	MDA‐MB‐361
									NTDSPLRY*	MDA‐MB‐453
									NTDSPLRY*	MCF7

*Note*: Peptides showing partial match with known T‐cell epitopes are marked with an asterisk (*). Epitope IDs are the reference number of T‐cell epitope listed on Tantigen (IDs beginning with T) and IEDB (IDs beginning with number).

Additionally, the list of known T‐cell epitope sequences identified in the EV ligandome includes peptides derived from antigens which are either associated with cancer or used as diagnostic and prognostic biomarkers. For example, peptides KTINPQVSKTEY and TYLPTNASLSF detected in the EV ligandomes of MDA‐MB‐415 and HCC 1954, respectively, are known T‐cell epitopes from Mammaglobin‐A and Her‐2, and thus relevant to breast cancer. Similarly, the peptide STAPPAHGV identified in the EV ligandome of myeloma cell line RPMI 8226, is a T‐cell epitope of MUC1 which has been identified as a tumour antigen in several multiple myeloma cell lines [28, 29, 30]. These results show for the first time that EVs released by cancer cells frequently carry T‐cell epitope peptides which may be clinically relevant to cancer.

The cell and EV ligandomes of all cell lines were also searched for the presence of additional peptides (TAApep) derived from TAA proteins. We predicted these would be present in our ligandomes because of the extended HLA‐I allele cohort present in our study, such that peptides derived by antigen processing of other parts of the TAA would likely be detected. We used Tantigen 2.0 database to identify peptides derived from the full‐length protein sequences of TAA. A total of 277 additional TAApep derived from 107 TAA proteins were identified in cell ligandomes of all lines (File S4). Out of the 277 TAApep, 39 TAApep were shared 45 times between different cell lines (representing a total of 322 TAApep; 5.2% of cell ligandome; Table 1, Column 6 and File S4). In EV ligandomes, 122 TAApep from 68 TAA proteins were identified (File S4). Of the 122 TAApep identified in the EV ligandome, 9 TAApep were shared amongst different EV ligandomes (total 131 TAApep; 5.4% of EV ligandome, Table 2, Column 6 an, File S4). A shortlist of TAApep identified in EV ligandomes is presented in Table 4 which contains TAApep derived from TAA proteins which have at least one defined T‐cell epitope in Tantigen database.

**TABLE 4:** Shortlist of peptides derived from TAA proteins (TAApep) detected in EV HLA‐I ligandome of breast cancer, melanoma, and myeloma cell lines

Number	Antigen full name	Antigen name	Antigen accession	Protein accession	Uniprot protein entry	Number of peptides	Peptide	Cell lines
1	Canalicular multispecific organic anion transporter 2	ABCC3	Ag000410	O15438	MRP3_HUMAN	2	AEKAFVSV	HCC 1395
							AYLHTTTTF	MDA‐MB‐361
2	Ataxin‐2‐like protein	ATXN2L	Ag004315	Q8WWM7	ATX2L_HUMAN	1	AHYPSQPVF	HCC 1806, RPMI 8226 8226
3	G1/S‐specific cyclin‐D1	CCND1	Ag000285	P24385	CCND1_HUMAN	4	NYLDRFLSL	HCC 1954
							AEETCAPSV	HCC 1395
							EVFPLAMNY	ESTDAB‐056
							EVFPLAMNYL	ESTDAB‐056
4	Cell division control protein 45 homologue	CDC45	Ag004342	O75419	CDC45_HUMAN	1	RPVNVVNVY	HCC 1954
5	Cleavage and polyadenylation specificity factor subunit 1	CPSF1	Ag000125	Q10570	CPSF1_HUMAN	2	SVLPAYLSY	HCC 1395
							ETVSGLKGY	ESTDAB‐056
6	Catenin beta‐1	CTNNB1	Ag000058	P35222	CTNB1_HUMAN	2	HPPSHWPLI	HCC 1806
							AQNAVRLHY	HCC 1806
7	Receptor tyrosine‐protein kinase erbB‐2	ERBB2	Ag000001	P04626	ERBB2_HUMAN	2	TPTAENPEY	HCC 1954
							MPNPEGRYTF	HCC 1954
8	ets variant 5	ETV5	Ag004221	P41161	ETV5_HUMAN	1	KVAGERYVY	ESTDAB‐056
9	Neutral alpha‐glucosidase AB	GANAB	Ag004500	Q14697	GANAB_HUMAN	1	AVAAVAARR	HCC 1806
10	Glycoprotein NMB	GPNMB	Ag000250	Q14956	GPNMB_HUMAN	1	STINYKWSF	ESTDAB‐056
11	Transcription factor HIVEP2	HIVEP2	Ag004270	P31629	ZEP2_HUMAN	1	SPLIRSNSV	HCC 1395
12	Heme oxygenase (decycling) 1	HMOX1	Ag000461	P09601	HMOX1_HUMAN	1	EVIPYTPAM	ESTDAB‐056
13	Heterogeneous nuclear ribonucleoprotein L	HNRNPL	Ag000394	P14866	HNRPL_HUMAN	2	VEFDSVQSA	HCC 1954
							IYIAGHPAF	HCC 1806
14	Heat shock 70 kDa protein 1B	HSPA1B	Ag000092	P0DMV9	HS71B_HUMAN	2	KQTQIFTTY	HCC 1806
							TVFDAKRLIGR	HCC 1806
15	Heat shock protein beta‐1	HSPB1	Ag004328	P04792	HSPB1_HUMAN	1	NEITIPVTF	MDA‐MB‐415
16	Insulin‐like growth factor 2 mRNA binding protein 3	IGF2BP3	Ag000506	O00425	IF2B3_HUMAN	1	ETAVVNVTY	ESTDAB‐056
17	Microtubule‐Actin cross‐linking factor 1 isoforms 1/2/3/5	MACF1	Ag004285	Q9UPN3	MACF1_HUMAN	3	EEAFHQGLISA	HCC 1395
							AEKFWYDMA	HCC 1395
							NQKPPSAEY	RPMI 8226 8226
18	Melanoma‐associated antigen C2	MAGEC2	Ag000036	Q9UBF1	MAGC2_HUMAN	1	NAVGVYAGR	HCC 1806
19	E3 ubiquitin‐protein ligase Mdm2 (Fragment)	MDM2	Ag000287	Q00987	MDM2_HUMAN	1	DEVYQVTVY	MDA‐MB‐415
20	ATPase MORC2	MORC2	Ag004287	Q9Y6X9	MORC2_HUMAN	1	IETELIYKY	MDA‐MB‐415
21	Nucleolar Protein Interacting With The FHA Domain Of MKI67	NIFK	Ag004290	Q9BYG3	MK67I_HUMAN	1	SQFGTVTRF	RPMI 8226 8226
22	2′‐5′‐oligoadenylate synthase 3	OAS3	Ag000403	Q9Y6K5	OAS3_HUMAN	2	TVLELVTQY	HCC 1954
							AEIISEIRA	HCC 1395 EV
23	Serine/threonine‐protein kinase PAK 2	PAK2	Ag000472	Q13177	PAK2_HUMAN	1	NENPLRALY	MDA‐MB‐415
24	Protein mono‐ADP‐ribosyltransferase PARP12	PARP12	Ag000523	Q9H0J9	PAR12_HUMAN	2	EYQKVWNLF	HCC 1954
							DEFGSWQEY	MDA‐MB‐415
25	Poly(rC)‐binding protein 2 (Fragment)	PCBP2	Ag004321	Q15366	PCBP2_HUMAN	1	LEGPPLEAY	MDA‐MB‐415
26	Serine/threonine‐protein phosphatase PP1‐alpha catalytic subunit	PPP1CA	Ag004293	P62136	PP1A_HUMAN	1	KYPENFFLL	HCC 1954
27	Replication protein A 70 kDa DNA‐binding subunit (Fragment)	RPA1	Ag000443	P27694	RFA1_HUMAN	2	AEAILGQNAA	HCC 1395
							KVIDQQNGLY	MDA‐MB‐415
28	Protein SON	SON	Ag004333	P18583	SON_HUMAN	3	SAYERSMM	HCC 1395
							YTDSYTDTY	MDA‐MB‐453
							SPMAERSMM	ESTDAB‐056
29	Serine/Arginine Repetitive Matrix 2	SRRM2	Ag004276	Q9UQ35	SRRM2_HUMAN	1	SPRKPIDSL	ESTDAB‐056
30	Signal transducer and activator of transcription	STAT1	Ag000451	P42224	STAT1_HUMAN	3	EELEQKYTY	MDA‐MB‐415
							SEVLSWQF	MDA‐MB‐415
							DQYSRFSL	MDA‐MB‐415
31	STAGA complex 65 subunit gamma	SUPT7L	Ag000225	O94864	ST65G_HUMAN	1	GSSPVFNQR	HCC 1806
32	Tensin‐3	TNS3	Ag004302	Q68CZ2	TENS3_HUMAN	3	YITERIIAV	HCC 1395
							QQMVVAHQY	MDA‐MB‐415
							TERIIAVSF	MDA‐MB‐415
33	DNA topoisomerase 2‐alpha	TOP2A	Ag000436	P11388	TOP2A_HUMAN	1	ATKTKFTM	HCC 1395
34	Topoisomerase (DNA) II beta 180 kDa	TOP2B	Ag000441	Q02880	TOP2B_HUMAN	1	SPRYIFTML	ESTDAB‐056
35	Targeting protein for Xklp2	TPX2	Ag004304	Q9ULW0	TPX2_HUMAN	1	KSSDQPLTV	HCC 1954
36	Thymidylate synthase	TYMS	Ag000483	P04818	TYSY_HUMAN	1	DAHIYLNHI	HCC 1806
37	Serine/threonine‐protein kinase WNK2 (Fragment)	WNK2	Ag000399	Q9Y3S1	WNK2_HUMAN	2	HESDVKIVA	HCC 1954
							QEHVPTSSA	HCC 1954
38	Zinc Finger CCCH‐Type Containing 14	ZC3H14	Ag004311	Q6PJT7	ZC3HE_HUMAN	1	KTTNVRQTY	ESTDAB‐056

*Note*: The shortlist is selected for TAAs which have at least one immunogenic peptide defined in Tantigen database.

In summary, our data indicate that the EV ligandomes of common cancer cell lines spanning breast, melanoma, and myeloma carry peptides from known TAAs, potential T‐cell epitopes, and additional TAApep derived from the protein sequences of known and established TAA.

## DISCUSSION

In this study, we report the first direct evidence of known tumour‐associated antigens and T‐cell epitopes presented on HLA‐I molecules on extracellular vesicles released by cancer cell lines. We have analysed the EV HLA‐I ligandomes of 10 cell lines, predominately representing breast cancer, but also including melanoma and myeloma to indicate that the presence of HLA‐I associated TAA on EV is likely to be a widespread observation and not exclusive to just one cancer type.

In recent years, EVs have emerged as promising tools in both cancer diagnosis and cancer immunotherapy [31]. EVs represent a cell‐free system for direct delivery of immune relevant peptides in association with HLA molecules [16, 17]. EVs derived from different cells have already been used in clinical trials; however, the majority of early trials have displayed only modest success in stimulation of an anti‐tumour response in effector T cells [32, 33]. Therefore, the challenge of selecting suitable target antigens that could be used for effective vaccine development remains. Use of EVs as cell free vaccines with preloaded antigen requires existing knowledge about the antigen [16]. Identifying EV HLA‐I ligandome of cancer cells offers the opportunity to study and identify complex peptidome to potentially gain insight of overall immuno‐peptidome of the EVs as well as the selection of target antigens. Moreover, identification of target antigens from EVs can be relatively non‐invasive since EVs can be collected from several body fluids. For example, EVs derived from ascites or malignant effusions have been used in in the immunotherapy of colorectal cancer [34].

Despite the clinical relevance and therapeutic potential of EVs, the EV HLA‐I ligandome of tumour cells has not been established in detail. To date, only two reports of the HLA‐I EV ligandome of cell lines have been published [35]. This low level of reporting on the HLA‐I ligandome is most likely due to the challenges of obtaining sufficient EV source material for mass spectrometry‐based sequencing of HLA‐I peptides. Nevertheless, our results show that despite low expression of HLA‐I molecules on EVs released by several of the breast cancer cell lines, it is still possible to identify tumour antigenic peptides and thus potential T‐cell epitopes. For example, HLA‐I expression was not detected by HC10 immunoblot analysis in EVs of cell lines MCF7, MDA‐MB‐231, and MDA‐MB‐361 (Figure 1c), yet tumour antigen peptides with known T‐cell activity were identified.

We have identified peptides in HLA‐I ligandomes of 10 cancer cell line. The peptide repertoire reported here may be used as database to identify multiple markers for diagnosis, prognosis of tumour as well as targets for immunotherapy. Interestingly, 26% of peptides present in EV HLA‐I ligandome were not found in HLA‐I ligandome of cells. The presence of unique peptides in HLA‐I ligandomes was also detected in previous two studies of Jesthom and JY cells [18, 35]. The reasons for this discrepancy is not yet understood, and may be a technical issue related to the amounts of input material, whereby much larger‐scale purification of cells and EV would result in better overlaps of cell and EV ligandomes. However, it remains a possibility that the cell and EV ligandomes do not fully overlap due to the biogenesis of the exosome component of the EV pool having originated in the endosomal/MVB pathway, where the possibility of peptide exchange may occur, generating new antigens in the EV ligandome [36]. Of some significance, the constituent 8‐15‐mer peptides of HLA‐I EV ligandomes of all cell lines contained 150 peptides representing both known T‐cell epitopes and additional TAApep derived from TAA proteins. Furthermore, the analysis settings of the mass spectrometry data we have used in this study are of relatively high stringency, and therefore a wider range of antigenic peptides of interest may be present and revealed by other analysis methodologies.

The presence of cell surface antigenic peptides presented on HLA‐I derived from TAA proteins, be it from differentiation antigens, over‐expressed cellular antigens, cancer/testis antigens or cancer related viral antigens allows the immune system the opportunity to mount responses that can impact on the progression of cancer growth [37]. Their presence on EV, as reported here, raises several interesting questions. Firstly, if HLA‐I TAA on EV were released by cancer cells into the bloodstream, they could potentially act as diagnostic, prognostic, and treatment monitoring biomarkers for cancer. Low recovery and heterogeneity of EVs by current isolation methods has largely restricted the detection of lowly expressed EV molecules. However, methods are available for the rapid enrichment of EV from serum or plasma by simple size exclusion columns [38, 39], and the EV can then be processed by our techniques herein to reveal the EV HLA‐I ligandome. Potentially, cancer‐specific EV could be enriched even further by monoclonal antibody‐affinity‐based methods [40], thus isolating only those EV released by cancer cells, allowing the characterization of the cancer‐specific EV HLA‐I ligandome occurring in that patient. Second, the presence of HLA‐I‐associated TAA on EV released by cancer cells could potentially represent a novel immune‐evasion strategy. Cancer specific CD8^+^ T cells approaching a tumour site could theoretically encounter a ‘cloud’ or gradient of EV containing HLA‐I presented TAA that could activate the T cell off‐target. There have, to date, been very few studies on the ability of HLA‐I on EV to activate CD8^+^ T cells, but EV loaded in vitro with common antigenic viral peptides can stimulate IFNγ secretion in purified CD8+ T cells, thus indicating that EV can indeed directly activate T cells [41].

Studies of patients undergoing monoclonal antibody checkpoint inhibitor therapy, which unleashes wider T cell activity, reveal that the best responses occur against tumours that typically have increased numbers of mutations, that is, which have the potential to generate large numbers of neo‐epitopes [42]. In recent years, it has become clear that the range of peptide sources for presentation by HLA‐I molecules is far wider than just from traditional proteins, and now encompasses non‐canonical sources such as from mis‐translations of normal mRNA, non‐coding RNA, and in addition cis‐ and trans‐peptide splicing events that occur in the proteasome [37]. At present, it is not known to what extent these sources of HLA‐I peptides contribute to the EV HLA‐I ligandome. However, we propose that the EV HLA‐I ligandome may provide a rich source for the identification of clinically relevant antigenic peptides in both health and disease from a relatively easy to access biofluids.

## CONFLICTS OF INTEREST

The authors declare no conflicts of interest.

## AUTHOR CONTRIBUTIONS

Simon J. Powis and Sally Shirran conceived the research plan. Pankaj Kumar, Simon J. Powis, and Sally Shirran designed the experiments. Pankaj Kumar, Simon J. Powis, Caitlin Boyne, Sydney Brown, and Ayesha Qureshi performed experiments. Sally Shirran and Silvia A. Synowsky performed mass spectrometry analysis of samples. PT identified HLA type of cell lines. Pankaj Kumar, Caitlin Boyne, Sydney Brown, and Simon J. Powis analysed data. Pankaj Kumar, Simon J. Powis, and Caitlin Boyne wrote manuscript.

## Supporting information

Supporting information

**TFile S1**Containing details of HLA genotypes of cell lines used in the study.

**TFile S2**Containing details of HLA‐I peptides identified by PEAKS DB search from cell and EV lysates of each cell line

**File S3**Containing details of cell and EV ligandomes and their comparisons

**File S4**TAApep identified in EVs of breast cancer, melanoma and myeloma cell lines

## Data Availability

Mass spectrometry proteomic data have been deposited to the ProteomeXchange Consortium via the PRoteomics IDEntifications (PRIDE) repository which can be accessed by reviewers (PRIDE dataset identifier: PXD025345).
