# Supplementary material for: Tumour‐associated antigenic peptides are present in the HLA class I ligandome of cancer cell line derived extracellular vesicles
Source: Immunology. 2022 Apr 20;166(2):249–64. doi: 10.1111/imm.13471 (PMC10357481; doi:10.1111/imm.13471)
Supplement: Supplementary file 1 — Supporting information [file IMM-166-249-s003.docx]

*Supporting information Figure S1.*

Comparison of cell and EV ligandomes. Cell and EV ligandomes were compared with each other to identify proportion of common peptides. a, comparison of cell and EV ligandomes of all cancer cell lines. b, comparison of cell and EV ligandomes of breast cancer cell lines. c, comparison of cell and EV ligandomes of melanoma cell line, ESTDAB-056. d, comparison of cell and EV ligandomes of myeloma cell line, RPMI 8226.


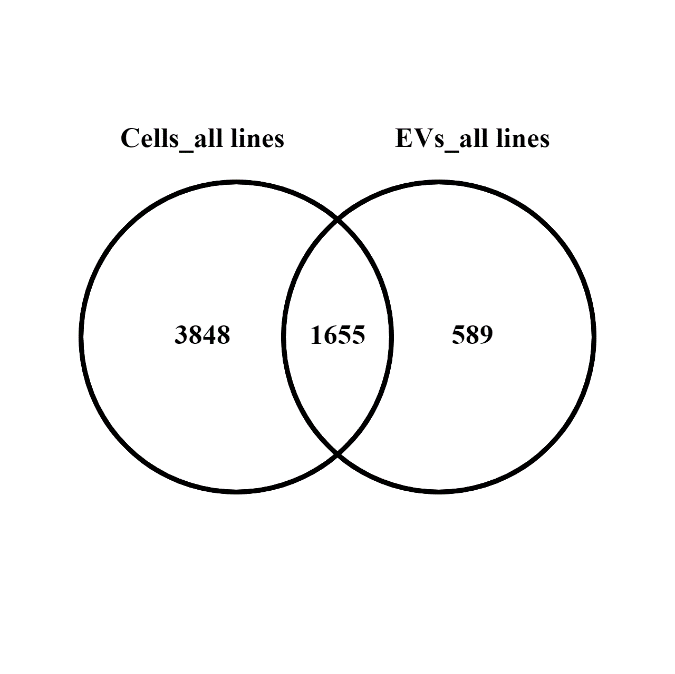

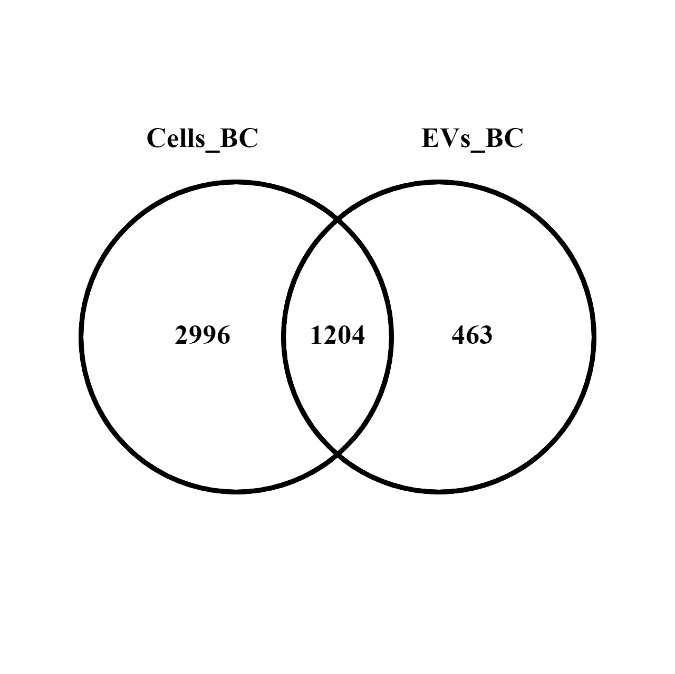

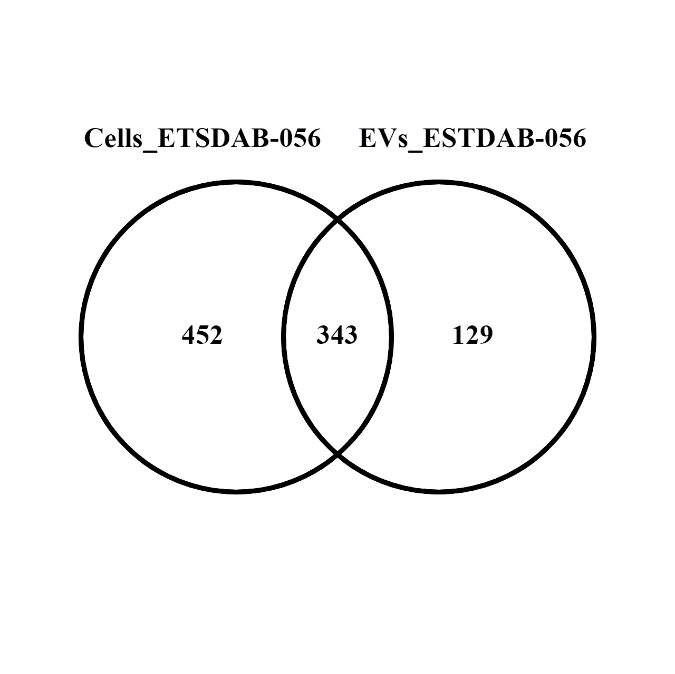

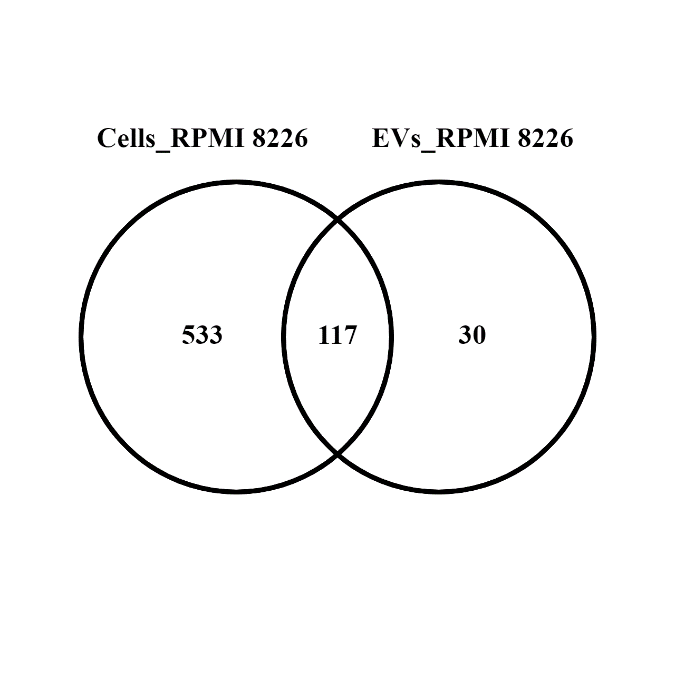


b

a

d

c

*Supporting information Figure S2.*

Predicted HLA-C binding affinities of peptides from cells (CL) and EV, determined using algorithm netMHCpan4.0. Each dot represents a single identified peptide. The numbers above each plot indicates the number of ligands for each respective HLA-I allele. Two-tailed Mann-Whitney were performed, with mean predicted affinity ± S.E. shown in red. ns= not significant; *= P<0.05.


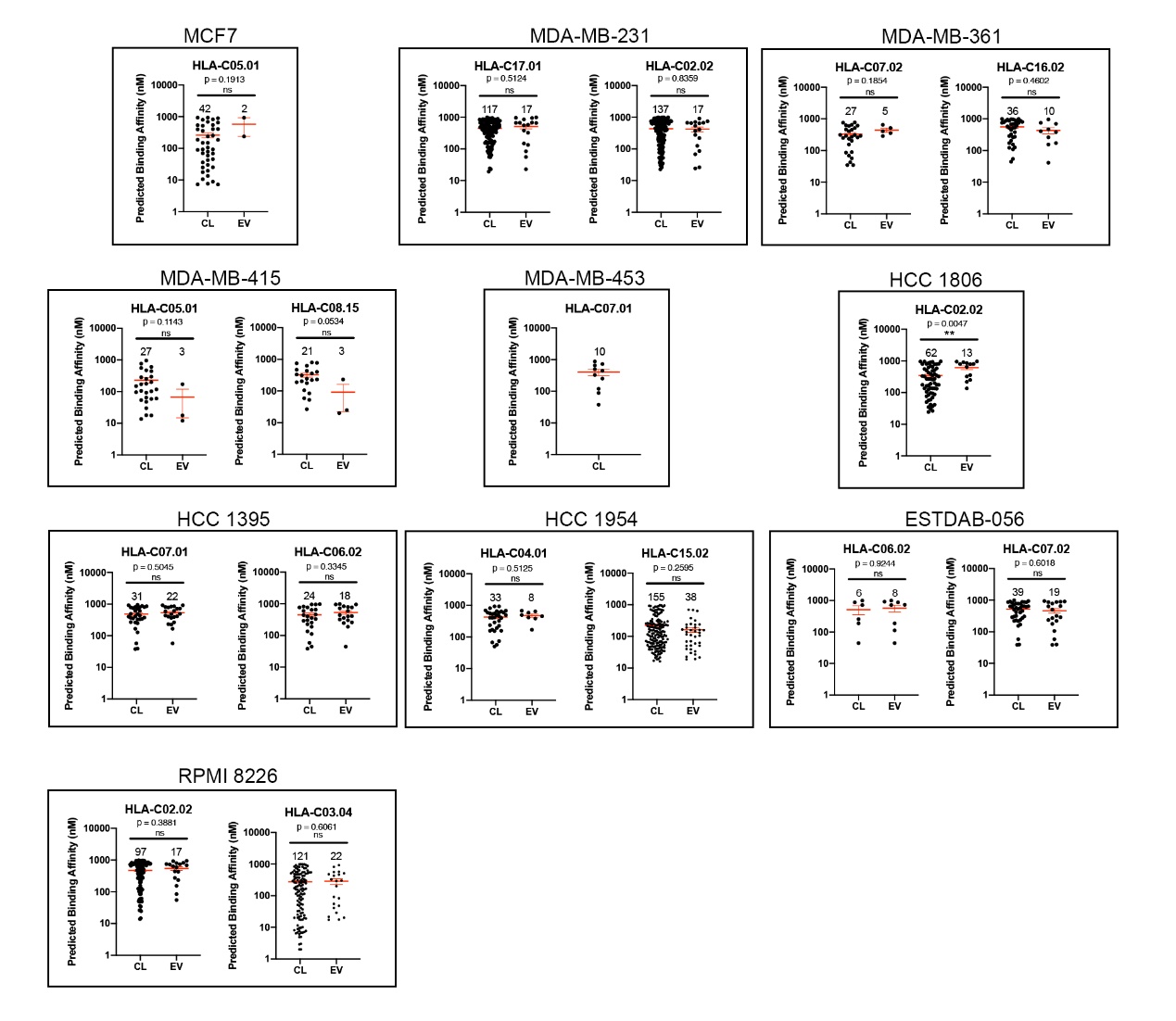


*Supporting information Table S1. Mean values of predicted binding affinities using netMHCpan4.0 of for HLA-A, –B and -C alleles from cell surface and EV ligandomes.*

| **Cell line** | **HLA-I allele** | **Cell lysate** | | **EV lysate** | |
| --- | --- | --- | --- | --- | --- |
|  |  | **Total number of HLA-I binding peptides** | **Mean of predicted HLA-I binding affinities** | **Total number of HLA-I binding peptides** | **Mean of predicted HLA-I binding affinities** |
| **MCF7** | HLA-A02.01 | 136 | 44.39 | 4 | 18.4 |
|  | HLA-B44.02 | 39 | 224.7 | 0 | no binding peptide |
|  | HLA-B18.01 | 68 | 180.28 | 0 | no binding peptide |
|  | HLA-C05.01 | 42 | 261.97 | 2 | 579 |
| **MDA-MB-231** | HLA-A02.17 | 234 | 238.81 | 26 | 203.04 |
|  | HLA-A02.01 | 321 | 83.56 | 28 | 48.99 |
|  | HLA-B41.01 | 34 | 246.89 | 4 | 320.73 |
|  | HLA-B40.02 | 39 | 215.12 | 5 | 572.06 |
|  | HLA-C17.01 | 117 | 449.78 | 17 | 511.39 |
|  | HLA-C02.02 | 137 | 432.75 | 17 | 420.17 |
| **MDA-MB-361** | HLA-A01.01 | 89 | 56.32 | 23 | 57.03 |
|  | HLA-A24.02 | 30 | 115.98 | 7 | 104.73 |
|  | HLA-B39.06 | 1 | 185.3 | 0 | no binding peptide |
|  | HLA-C07.02 | 27 | 327.07 | 5 | 441.62 |
|  | HLA-C16.02 | 36 | 556.69 | 10 | 429.52 |
| **MDA-MB-415** | HLA-A30.02 | 108 | 171.67 | 57 | 134.24 |
|  | HLA-A33.01 | 18 | 125.88 | 14 | 54.59 |
|  | HLA-B14.02 | 52 | 384.16 | 24 | 401.78 |
|  | HLA-B18.01 | 513 | 154.38 | 184 | 155.23 |
|  | HLA-C05.01 | 27 | 230.13 | 3 | 67.3 |
|  | HLA-C08.15 | 21 | 326.8 | 3 | 92.5 |
| **MDA-MB-453** | HLA-A01.01 | 330 | 48.47 | 82 | 28.91 |
|  | HLA-B08.01 | 118 | 158.24 | 33 | 130.25 |
|  | HLA-C07.01 | 10 | 402.99 | 0 | no binding peptide |
| **HCC 1806** | HLA-A68.01 | 207 | 112.18 | 104 | 60.85 |
|  | HLA-A23.01 | 135 | 118.28 | 50 | 60.91 |
|  | HLA-B51.01 | 51 | 295.49 | 26 | 381.52 |
|  | HLA-B15.03 | 334 | 123.95 | 152 | 86.56 |
|  | HLA-C02.02 | 62 | 346.27 | 13 | 615.45 |
| **HCC 1395** | HLA-A29.02 | 149 | 59.11 | 92 | 37.3 |
|  | HLA-B08.01 | 298 | 165.32 | 206 | 161.31 |
|  | HLA-B45.01 | 151 | 128.92 | 198 | 106.8 |
|  | HLA-C07.01 | 31 | 485.11 | 22 | 532.96 |
|  | HLA-C06.02 | 24 | 448.4 | 18 | 531.18 |
| **HCC 1954** | HLA-A24.02 | 154 | 85.71 | 75 | 88.42 |
|  | HLA-B35.01 | 197 | 77.11 | 81 | 71.84 |
|  | HLA-B40.06 | 217 | 219.47 | 102 | 221.54 |
|  | HLA-C04.01 | 33 | 423.87 | 8 | 478.19 |
|  | HLA-C15.02 | 155 | 224.96 | 38 | 163.18 |
| **ESTDAB-056** | HLA-A26.01 | 322 | 129.49 | 178 | 93.38 |
|  | HLA-A32.01 | 166 | 210.22 | 103 | 210.57 |
|  | HLA-B07.02 | 185 | 51.02 | 127 | 34.33 |
|  | HLA-B57.02 | 153 | 236.82 | 106 | 221.67 |
|  | HLA-C06.02 | 6 | 513.13 | 8 | 574.05 |
|  | HLA-C07.02 | 39 | 520.17 | 19 | 463.87 |
| **RPMI 8226** | HLA-A30.01 | 23 | 291.03 | 4 | 58.4 |
|  | HLA-A68.02 | 110 | 81.79 | 21 | 24.49 |
|  | HLA-B15.03 | 431 | 150.99 | 81 | 149.46 |
|  | HLA-B15.10 | 133 | 354.33 | 28 | 364.1 |
|  | HLA-C02.02 | 97 | 468.46 | 17 | 544.95 |
|  | HLA-C03.04 | 121 | 276.41 | 22 | 286.21 |

*Supporting information Table S2. Summary of cell and EV ligandome of breast cancer, melanoma and myeloma cell lines after removing duplicate peptides present in more than one cell line or EV sample.*

| **Cell lysate** | | | | | | **EV lysate** | | | | | |
| --- | --- | --- | --- | --- | --- | --- | --- | --- | --- | --- | --- |
| **1. Cell Lines** | **2. Total number of eluted peptides** | **3. Number of peptides in HLA-I ligandome (8-15-mer peptides)** | **4. Number of netMHCpan 4.0 binding peptides** | **5. Number of known immunogenic peptides identified in the study** | **6. Number of peptides derived from known TAA (TAApep)** | **1. Cell Lines** | **2. Total number of eluted peptides** | **3. Number of peptides in HLA-I ligandome (8-15-mer peptides)** | **4. Number of netMHCpan 4.0 binding peptides** | **5. Number of known immunogenic peptides identified in the study** | **6. Number of peptides derived from known TAA (TAApep)** |
| Total | 6574 | 6144 | 5116 | 43 | 322 |  | 2461 | 2406 | 2108 | 19 | 131 |
| Total breast cancer | 5105 | 4699 | 3819 | 38 | 245 |  | 1839 | 1787 | 1563 | 15 | 101 |

*Supporting information Table S3. List of peptides matching with previously identified T-cell epitopes from cell surface HLA-I ligandomes of breast cancer, melanoma and myeloma cell lines. Peptides showing partial match with known T-cell epitopes are marked with an asterisk (*). Epitope IDs are the reference number of T-cell epitope listed on Tantigen (IDs beginning with T) and IEDB (IDs beginning with number).*

| **Number** | **Antigen name** | **Parent protein** | **Epitope ID** | **Position** | **Uniprot entry name** | **PubMed ID** | **T-cell epitope** | **HLA -I restriction** | **Peptides identified in the study** | **Cell Line** |
| --- | --- | --- | --- | --- | --- | --- | --- | --- | --- | --- |
| 1 | Fructose-bisphosphate aldolase A | P04075 | 2874 | 216-224 | ALDOA_HUMAN | 11782012 | ALSDHHIYL | HLA-A*02:01 | ALSDHHIYL | MCF7 |
| 2 | Clathrin heavy chain 1 | Q00610 | 442923 | 311-320 | CLH1_HUMAN | 22869377 | ATAGIIGVNR | HLA-A*11 | ATAGIIGVNR | HCC 1806 |
| 3 | E3 ubiquitin-protein ligase Mdm2 | Q00987 | 236802 | 273-281 | MDM2_HUMAN | 25548167 | DEVYQVTVY | HLA-B*18 | DEVYQVTVY | MCF7 |
|  |  |  |  |  |  |  |  |  | DEVYQVTVY | MDA-MB-415 |
| 4 | Histone H3.3 | P84243 | T000946 | 59-67 | H33_HUMAN | 16196104 | ELLIRKLPF | HLA-B*8 | ELLIRKLPF | HCC 1395 |
|  |  |  |  |  |  |  |  |  | ELLIRKLPF | MDA-MB-453 |
| 5 | G1/S-specific cyclin-D1 | P24385 | T000697 | 115-124 | CCND1_HUMAN | 12384544 | ETIPLTAEKL | HLA-A*68:01 | ETIPLTAEKL | HCC 1806 |
| 6 | Cytochrome P450 1B1 | Q16678 | T000701 | 5-13 | CP1B1_HUMAN | 12869499 | FLDPRPLTV | HLA-A*02:01 | FLDPRPLTV | MCF7 |
|  |  |  |  |  |  |  |  |  | FLDPRPLTV | MDA-MB-231 |
| 7 | Fatty acid synthase | P49327 | 16587 | 2335-2345 | FAS_HUMAN | 11782012 | FLFDGSPTYVL | HLA-A*02:01 | FLFDGSPTYVL | MDA-MB-231 |
| 8 | Melanoma-associated antigen E1 | Q9HCI5 | 505372 | 265-273 | MAGE1_HUMAN | 29557506 | FVYGEPREL | HLA-A*02:01 | FVYGEPREL | RPMI 8226 |
| 9 | DNA (cytosine-5)-methyltransferase 1 | P26358 | 20914 | 425-433 | DNMT1_HUMAN | 15382068 | GLIEKNIEL | HLA-A*2 | GLIEKNIEL | MCF7 |
|  |  |  |  |  |  |  |  |  | GLIEKNIEL | MDA-MB-231 |
| 10 | Catenin beta-1 | P35222 | 20996 | 400-408 | CTNB1_HUMAN | 11782012 | GLLGTLVQL | HLA-A*02:01 | GLLGTLVQL | MCF7 |
|  |  |  |  |  |  |  |  |  | GLLGTLVQL | MDA-MB-231 |
| 11 | Perilipin-2 | Q99541 | 237022 | 313-321 | PLIN2_HUMAN | 25548167 | IARNLTQQL | HLA-B*7 | IARNLTQQL | ESTDAB-056 |
| 12 | B-cell receptor-associated protein 31 | Q53G72 | T000888 | 167-175 | Q53G72_HUMAN | 12750359 | KLDVGNAEV | HLA-A*2 | KLDVGNAEV | MCF7 |
|  |  |  |  |  |  |  |  |  | KLDVGNAEV | MDA-MB-231 |
| 13 | Signal transducer and activator of transcription 1, 91kDa | P42224 | T000895 | 350-358 | STAT1_HUMAN | 12750359 | KLQELNYNL | HLA-A*2 | KLQELNYNL | MCF7 |
| 14 | Cyclin I | Q14094 | T000893 | 70-78 | CCNI_HUMAN | 12750359 | LLDRFLATV | HLA-A*2 | LLDRFLATV | MCF7 |
| 15 | Gamma-interferon-inducible lysosomal thiol reductase | P13284 | 37474 | 15-24 | GILT_HUMAN | 30115740 | LLLDVPTAAV | HLA-A*02:01 | LLDVPTAAV* | MCF7 |
| 16 | Histone deacetylase HD1 | Q13547 | 419900 | 373-381 | HDAC1_HUMAN | 27467910 | LPHAPGVQM | HLA-B*7 | MLPHAPGVQM* | MDA-MB-231 |
| 17 | Histone-binding protein RBBP4 | Q09028 | 215983 | 245-253 | RBBP4_HUMAN | 22869377 | NLKLKLHSF | HLA-B*57 | NLKLKLHSF | HCC 1395 |
|  |  |  |  |  |  |  |  |  | NLKLKLHSF | MDA-MB-453 |
| 18 | Septin 2 | Q15019 | T000894 | 256-265 | SEPT2_HUMAN | 12750359 | RLYPWGVVEV | HLA-A*2 | RLYPWGVVEV | MDA-MB-231 |
| 19 | AN1-type zinc finger protein 5 | O76080 | 237332 | 63-73 | ZFAN5_HUMAN | 25548167 | SASVQRADTSL | HLA-B*7 | SASVQRADTSL | ESTDAB-056 |
| 20 | Protein flightless-1 homolog | Q13045 | 59100 | 1010-1018 | FLII_HUMAN | 11782012 | SLFPGKLEV | HLA-A*02:01 | SLFPGKLEV | MDA-MB-231 |
| 21 | CD59 glycoprotein precursor | P13987 | 59453 | 106-114 | CD59_HUMAN | 11782012 | SLSEKTVLL | HLA-A*02:01 | SLSEKTVLL | MDA-MB-231 |
| 22 | Collagen alpha-5(IV) chain | P29400 | 59582 | 18-26 | CO4A5_HUMAN | 11782012 | SLWGQPAEA | HLA-A*02:01 | SLWGQPAEA | MCF7 |
|  |  |  |  |  |  |  |  |  | SLWGQPAEA | MDA-MB-231 |
| 23 | Chromatin Assembly Factor 1 Subunit A | Q13111 | T001077 | 772-781 | CAF1A_HUMAN | 24048523 | SPRSPSTTYL | HLA-B*0702 | SPRSPSTTYL | ESTDAB-056 |
| 24 | HNRPLL protein | Q8WVV9 | 120231 | 71-83 | HNRLL_HUMAN | 22869377 | SVSPVVHVR | HLA-A*11 | SVSPVVHVR | HCC 1806 |
| 25 | Mammaglobin-A precursor | Q13296 | 64399 | 32-40 | SG2A2_HUMAN | 15538043 | TINPQVSKT | HLA-A*2 | KTINPQVSKTEY* | MDA-MB-415 |
| 26 | Protein BTG1 | P62324 | 65151 | 103-111 | BTG1_HUMAN | 11782012 | TLWVDPYEV | HLA-A*02:01 | TLWVDPYEV | MCF7 |
|  |  |  |  |  |  |  |  |  | TLWVDPYEV | MDA-MB-231 |
| 27 | HER2 receptor | P04626 | 67385 | 63-71 | ERBB2_HUMAN | 17397516 | TYLPTNASL | HLA-A*24 | TYLPTNASLSF* | HCC 1954 |
|  |  |  |  |  |  |  |  |  | TYLPTNASLSF* | HCC 1806 |
| 28 | Guanine nucleotide-binding protein-like 3-like protein | Q9NVN8 | 418709 | 293-301 | GNL3L_HUMAN | 22869377 | VYLDKFIRL | HLA-A*24 | VYLDKFIRL | HCC 1806 |
| 29 | Poly (ADP-ribose) polymerase family, member 12 | Q9H0J9 | T000954 | 669-677 | PAR12_HUMAN | 16033845 | VYPEYVIQY | HLA-C*0702 | VYPEYVIQY | ESTDAB-056 |
| 30 | Ubiquitin-conjugating enzyme E2 D2 | P62837 | 243855 | 59-66 | UB2D2_HUMAN | 22869377 | YPFKPPKV | HLA-B*51 | YPFKPPKVAF* | HCC 1954 |
|  |  |  |  |  |  |  |  |  | YPFKPPKVTF* | HCC 1954 |
|  |  |  |  |  |  |  |  |  | YPFKPPKV | HCC 1806 |
| 31 | 40S ribosomal protein SA | P08865 | T000873 | 146-154 | RSSA_HUMAN | 16709854 | ALCNTDSPL | HLA-A*02:01 | NTDSPLRY* | MDA-MB-453 |
